# The impact of ABO blood groups and RhD status on systemic inflammation markers (Systemic Immune-Inflammation Index, Systemic Inflammation Response Index, and Pan-Immune-Inflammation Value) in a large cohort of healthy adults

**DOI:** 10.1093/labmed/lmag073

**Published:** 2026-10-26

**Authors:** Ali Osman AVCI

**Affiliations:** Department of Internal Medicine, Faculty of Medicine, Lokman Hekim University, Ankara, Türkiye

**Keywords:** ABO blood groups, RhD status, Systemic Immune-Inflammation Index (SII), Systemic Inflammation Response Index (SIRI), Pan-Immune-Inflammation Value (PIV), healthy cohort, sexual dimorphism

## Abstract

**Introduction:**

The Systemic Immune-Inflammation Index (SII), Systemic Inflammation Response Index (SIRI), and Pan-Immune-Inflammation Value (PIV) are composite, integrated biomarkers of host inflammatory status. Although ABO blood groups are linked to various systemic diseases, their influence on these novel indices remains unclear. This study evaluated the impact of ABO blood groups and Rh blood group, D antigen (RhD) status on SII, SIRI, and PIV in a rigorously screened healthy cohort.

**Methods:**

We retrospectively analyzed the electronic health records of 923 healthy adults (396 male, 527 female) evaluated between 2021 and 2025. Inclusion required strict metabolic (hemoglobin A_1c_ < 5.7%), organ function, and serologic stability. Multigroup comparisons across the 4 ABO blood groups were performed using the Kruskal-Wallis *H* test, whereas 2-group independent analyses for sex and RhD status were evaluated using the Mann-Whitney *U* test.

**Results:**

No statistically significant differences were observed in SII, SIRI, or PIV scores across the 4 major ABO blood groups (*P* = .61, *P* = .498, and *P* = .61, respectively) or RhD status (SII, *P* = .34; SIRI, *P* = .06; and PIV, *P* = .33). Statistically significant sex-based dimorphism was identified: SII scores were higher in female individuals (*P* = .007), whereas SIRI scores were higher in male individuals (*P* = .04). Chronological age categories showed no statistically significant correlation with any index (*P* > .05).

**Discussion:**

Systemic inflammatory indices are independent of ABO blood groups and RhD status in clinically healthy adults. The substantial sex-specific variations underscore the necessity of considering implementing sex-stratified reference intervals within laboratory information systems to enhance personalized clinical interpretation.

## Introduction

The clinical utility of systemic inflammation markers derived from routine complete blood cell count parameters has gained considerable prominence in contemporary medicine. Among these, the Systemic Immune-Inflammation Index (SII), Systemic Inflammation Response Index (SIRI), and Pan-Immune-Inflammation Value (PIV) have emerged as highly robust, cost-effective prognostic and diagnostic tools. Their clinical value has been widely validated across a broad spectrum of pathologies, ranging from acute cardiovascular disorders^1‐4^ to complex oncologic malignancies.^5‐7^ Unlike traditional single-cell ratios such as the neutrophil to lymphocyte ratio,^8^ these advanced composite indices concurrently integrate multiple leukocyte lineages and platelets. Consequently, they provide a far more comprehensive and nuanced reflection of the delicate balance between active systemic inflammation and host immune surveillance, uniquely redefining personalized stratification paradigms within therapeutic settings.^9^

The ABO blood group system, discovered by Karl Landsteiner, remains the cornerstone of transfusion medicine and immunohematology. However, emerging evidence suggests that ABO blood group antigens are also intricately linked to the pathogenesis of various chronic systemic and cardiovascular conditions.^10^ These carbohydrate antigens are not restricted to erythrocytes; they are highly expressed on vascular endothelial cells, platelets, and plasma glycoproteins. They modulate disease susceptibility by altering the clearance rates and plasma levels of von Willebrand factor and factor VIII and by influencing the glycosylation patterns of circulating proinflammatory cytokines via ABO-specific transferases.^11^^,^^12^

Intriguingly, although a few pioneer studies have attempted to define reference intervals for some white blood cell–based markers in specific populations,^13^ the potential confounding influence of ABO phenotypes and Rh blood group, D antigen (RhD) status on the basal scores of SII, SIRI, and PIV remains largely unexplored.^14^ Furthermore, well-documented sex-specific differences in immune responses^15^^,^^16^ underscore the necessity of verifying these biomarkers in a nonpathologic baseline state. Recent clinical investigations have successfully used these composite inflammatory metrics to risk-stratify transient metabolic states such as gestational diabetes^17^^,^^18^ or multisystemic chronic inflammatory conditions such as Behçet disease.^19^ Because the vast majority of the existing literature focuses on cohorts with active disease,^20^ the true physiologic impact of blood group antigens on these indices may be obscured by ongoing inflammatory cascades.

Establishing whether ABO phenotypes influence the basal inflammatory “tonus” is therefore crucial for determining precise biological reference ranges and accurately interpreting these markers in personalized clinical practice. To validate these baselines, large-scale epidemiologic reference frameworks and updates on pathophysiology must be carefully synthesized. To address this gap in the literature, the present study aimed to comprehensively investigate the impact of ABO blood groups and RhD status on SII, SIRI, and PIV scores within a large, rigorously screened cohort of metabolically and serologically healthy adults.

## Methods

### Ethical consideration

The study protocol was approved by the Lokman Hekim University Scientific Research Ethics Committee (January 26, 2026; meeting No. 2026/1, decision No. 2026/028). Due to the retrospective nature of the study, the requirement for informed consent was waived. The research was conducted in strict accordance with the principles of the Declaration of Helsinki.

### Study population and selection criteria

This retrospective, cross-sectional study analyzed the electronic health records of individuals who had undergone routine health screenings at a tertiary-care center between January 2021 and December 2025. To establish a strictly “healthy cohort,” a rigorous multistage screening process was implemented. Inclusion was restricted to asymptomatic individuals who met the following stringent criteria:

- **Organ function and endocrine status:** Normal kidney function (creatinine <1.2 mg/dL),^20^ hepatic function (aspartate aminotransferase/alanine aminotransferase within reference ranges), and euthyroid status (thyrotropin [previously thyroid-stimulating hormone], 0.4-4.0 mIU/L; thyroxine, 0.80-1.80 ng/dL)
- **Metabolic and lipid profile:** Fasting blood glucose below 100 mg/dL and hemoglobin A_1c_ below 5.7%,^21^ total cholesterol below 200 mg/dL, and triglycerides below 200 mg/dL
- **Hematologic and serologic stability:** Hemoglobin levels of 12.0 to 16.0 g/dL for female individuals and 13.0 to 17.0 g/dL for male individuals^22^; total white blood cell count of 4.0 to 10.0 × 10^9^/L; and confirmed negative serology for hepatitis B surface antigen, anti–hepatitis C virus, and anti-HIV

Exclusion criteria were (1) history of malignancy or chronic autoimmune/inflammatory disorders, (2) evidence of active infection, (3) use of medications that modulate inflammatory responses (eg, nonsteroidal anti-inflammatory drugs, corticosteroids, statins, or hormone therapies), and (4) pregnancy.

### Laboratory procedures and index calculations

Venous blood samples were collected after an overnight fast. Complete blood cell counts were performed using K2-EDTA tubes, and biochemical assays were conducted using gel-separator tubes. All novel indices were derived from absolute cell counts (cells/mm^3^) using the following formulas:

- SII: (platelets × neutrophils) / lymphocytes
- SIRI: (neutrophils × monocytes) / lymphocytes
- PIV: (neutrophils × platelets × monocytes) / lymphocytes

Based on established reference literature in healthy adult cohorts, baseline physiologic reference medians generally range between 300 and 600 for SII, 0.8 and 1.5 for SIRI, and 200 and 400 for PIV, with scores above these thresholds indicating subclinical or systemic clinical inflammation.^3^^,^^13^^,^^19^^,^^23^

### Statistical analysis

Statistical analyses were performed using Python, version 3.13. Normality of data distribution was assessed using the Kolmogorov-Smirnov test.^24^ Because the systemic inflammatory indices (SII, SIRI, and PIV) exhibited a non-normal distribution, data are expressed as median (IQR). For multigroup comparisons across the 4 ABO blood groups, the Kruskal-Wallis *H* test was used. In contrast, for all 2-group independent comparisons—specifically, sex-based analyses (male vs female) and RhD status analyses (Rh positive vs Rh negative)—the Mann-Whitney *U* test was applied strictly to ensure methodologic consistency.

To further investigate the chronological age–related trends, the study population was stratified into 3 clinically relevant age categories: young adults (aged 18-39 years), middle-aged adults (aged 40-59 years), and older adults (aged ≥60 years). Differences across these categories were evaluated using the Kruskal-Wallis *H* test. Continuous correlation between chronological age and the indices was assessed using Spearman rank correlation. Post hoc statistical power analysis was performed to evaluate the reliability of our multigroup and subgroup findings. A 2-tailed *P* < .05 was considered statistically significant.

## Results

### Demographic distribution and cohort characteristics

The baseline demographic parameters, ABO blood group distributions, RhD phenotypes, and complete inflammatory index profiles stratified by sex and age categories are comprehensively summarized in Table 1. The final study cohort comprised 923 healthy adults: 396 (42.9%) male and 527 (57.1%) female, with an overall mean (SD) chronological age of 47.9 (14.2) years. The relative phenotypic distributions of the 4 major ABO blood groups and RhD status demonstrated a statistically balanced, homogeneous spread between both sexes (*P* = .61 and *P* = .81, respectively), verifying the absence of sex-based selection or administrative bias within the study population. These foundational demographic and phenotypic percentages across the cohort are graphically illustrated in the combined pie charts in Figure 1 (panel A for ABO distribution and panel B for RhD status).

**Figure 1:**
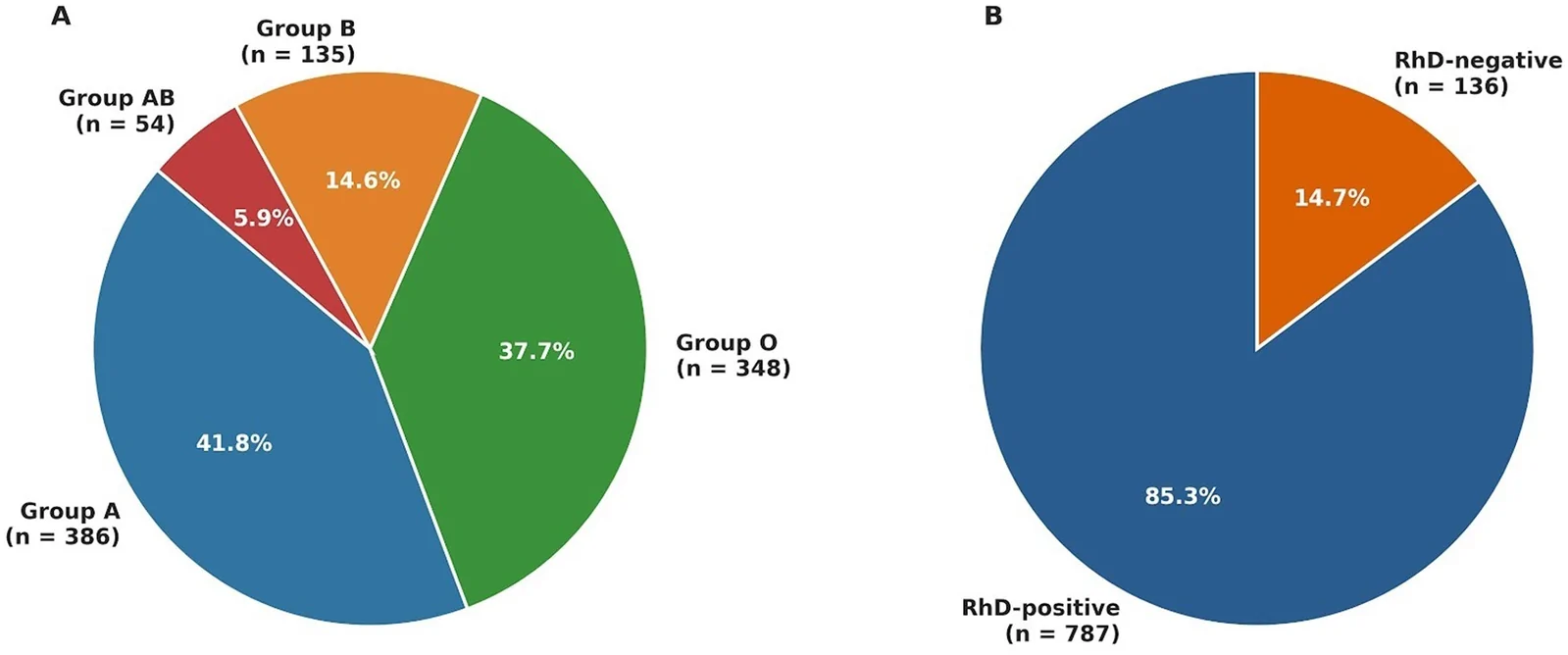
Baseline distribution of ABO blood groups and Rh blood group, D antigen (RhD) status across the healthy adult cohort (*n* = 923). **(A)** Pie chart illustrating the phenotypic proportion and absolute counts of the 4 main ABO blood groups within the screened population: group A (*n* = 386 [41.8%]), group O (*n* = 348 [37.7%]), group B (*n* = 135 [14.6%]), and group AB (*n* = 54 [5.9%]). **(B)** Pie chart depicting the independent percentage distribution of RhD status across the entire cohort: RhD positive (*n* = 787 [85.3%]) and RhD negative (*n* = 136 [14.7%]).

**Table 1:** Baseline patient demographic and hematologic characteristics, with stratified inflammatory indices.

Parameter	Total (*n* = 923)	Male (*n* = 396)	Female (*n* = 527)	*P* value
Age, mean (SD), y	47.9 (14.2)	48.2 (13.8)	47.7 (14.5)	.58^a^
ABO blood group, No. (%)				.61^b^
A	386 (41.8)	162 (40.9)	224 (42.5)	
B	135 (14.6)	58 (14.6)	77 (14.6)	
AB	54 (5.9)	23 (5.8)	31 (5.9)	
O	348 (37.7)	153 (38.6)	195 (37.0)	
RhD status, No. (%)				.81^a^
Positive	787 (85.3)	338 (85.4)	449 (85.2)	
Negative	136 (14.7)	58 (14.6)	78 (14.8)	
SII, median (IQR)	551.40 (395.00-798.00)	530.58 (380.70-756.80)	573.68 (407.00-835.30)	.007^c^
SIRI, median (IQR)	1.12 (0.70-1.70)	1.20 (0.75-1.82)	1.06 (0.73-1.53)	.04^c^
PIV, median (IQR)	289.20 (178.00-429.00)	280.71 (171.50-436.60)	279.94 (184.10-421.80)	.61^a^
Age subgroup, No. (%)				
18-39 y	302 (32.7)	128 (32.3)	174 (33.0)	
40-59 y	415 (45.0)	179 (45.2)	236 (44.8)	
≥60 y	206 (22.3)	89 (22.5)	117 (22.2)	

Abbreviations: PIV, Pan-Immune-Inflammation Value; RhD, Rh blood group, D antigen; SII, Systemic Immune-Inflammation Index; SIRI, Systemic Inflammation Response Index.

a Calculated using the independent 2-sample Mann-Whitney *U* test.

b Calculated using the multigroup Kruskal-Wallis *H* test. Statistically significant at *P* < .05.

c Spearman rank correlation analysis demonstrated no statistically significant correlation between chronological age and SII (*r* = −0.012, *P* = .72), SIRI (*r* = 0.031, *P* = .35), or PIV (*r* = −0.019, *P* = .56).

### Distribution of inflammatory indices across ABO and RhD blood groups

The absolute median scores and respective IQRs for SII, SIRI, and PIV were calculated for each blood group phenotype. No statistically significant differences or distinct phenotypic variations were observed in any of the integrated composite inflammatory indices across the 4 major ABO blood groups (Table 2):

**Table 2:** Comparison of SII, SIRI, and PIV across ABO blood groups.

Index	Group A, median (IQR) (*n* = 386)	Group B, median (IQR) (*n* = 135)	Group AB, median (IQR) (*n* = 54)	Group O, median (IQR) (*n* = 348)	*P* value^a^
SII	558.90 (407.00-801.00)	538.70 (375.00-741.00)	530.10 (369.00-787.00)	553.70 (396.00-773.00)	.61
SIRI	1.10 (0.75-1.63)	1.05 (0.65-1.64)	1.15 (0.80-1.53)	1.05 (0.73-1.64)	.498
PIV	279.20 (181.00-422.00)	281.20 (157.00-423.00)	302.30 (181.00-435.00)	268.60 (183.00-424.00)	.61

Abbreviations: PIV, Pan-Immune-Inflammation Value; SII, Systemic Immune-Inflammation Index; SIRI, Systemic Inflammation Response Index.

a *P* values calculated using the Kruskal-Wallis *H* test.

- **SII:** Median scores peaked in group A (558.9) and reached their lowest levels in group AB (530.1), demonstrating no statistical significance (*P* = .61). The box plot distribution across blood groups and RhD phenotypes is illustrated in Figure 2.
- **SIRI:** Median scores fluctuated minimally, ranging from 1.05 in groups B and O to 1.10 in group A and 1.15 in group AB, without reaching clinical or statistical significance (*P* = .498). The distribution variations are presented in Figure 3.
- **PIV:** Similarly, PIV scores remained highly consistent across all blood groups, yielding a median value of 268.6 in group O, 279.2 in group A, 281.2 in group B, and 302.3 in group AB (*P* = .61) (Table 2). The categorical spread is depicted in Figure 4.

**Figure 2:**
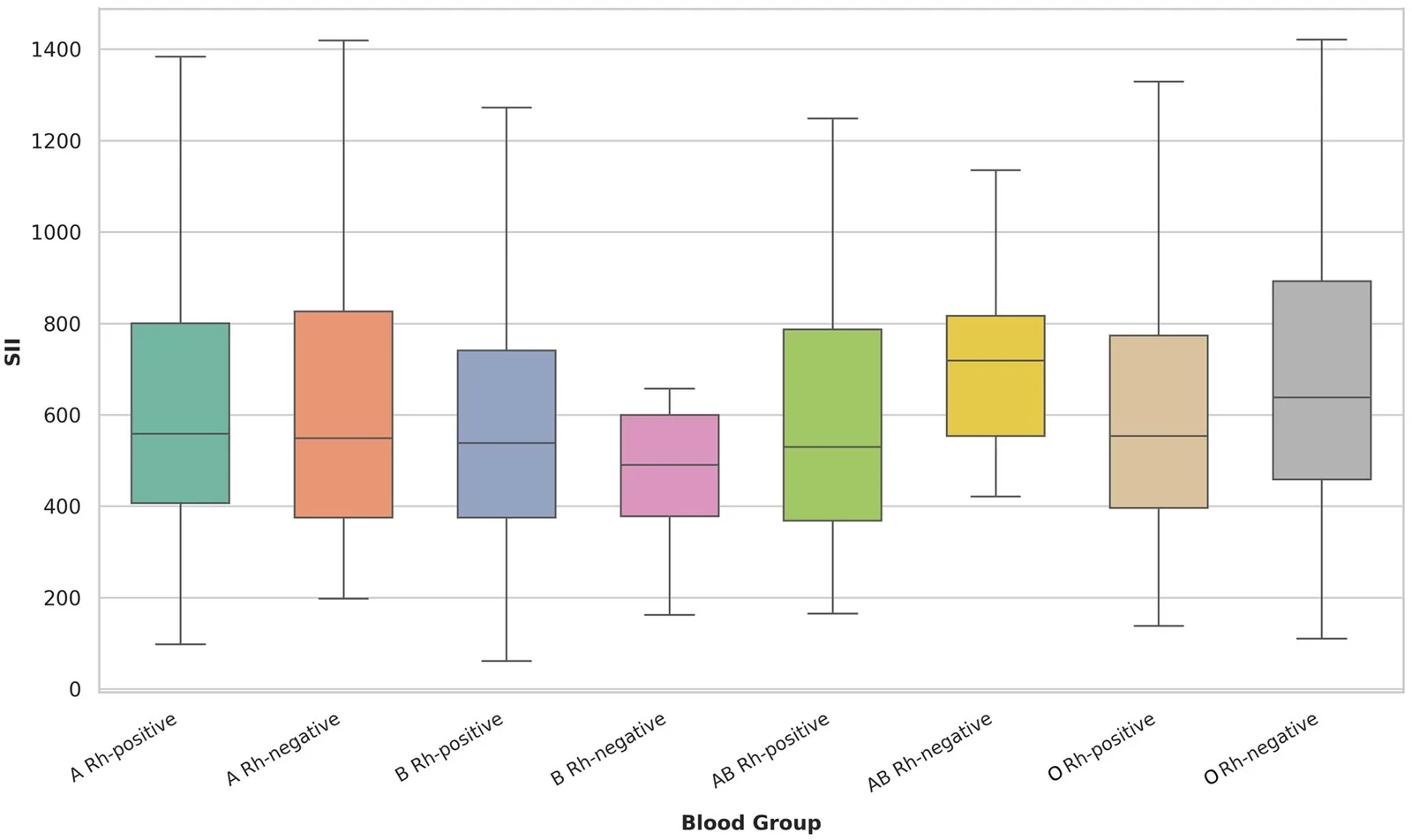
Distribution of the Systemic Immune-Inflammation Index (SII) across ABO and Rh blood group, D antigen blood group phenotypes. The box-and-whisker plot illustrates the baseline distribution of SII scores across the 8 standardized blood group combinations. The horizontal line within each box denotes the absolute median score, the box boundaries represent the IQR, and the whiskers represent the overall distribution range (outliers excluded). Statistical analysis by the Kruskal-Wallis *H* test indicated that baseline SII scores remain entirely independent of blood group phenotypes (*P* = .61). The index was calculated using the standardized formula (platelets × neutrophils) / lymphocytes.

**Figure 3:**
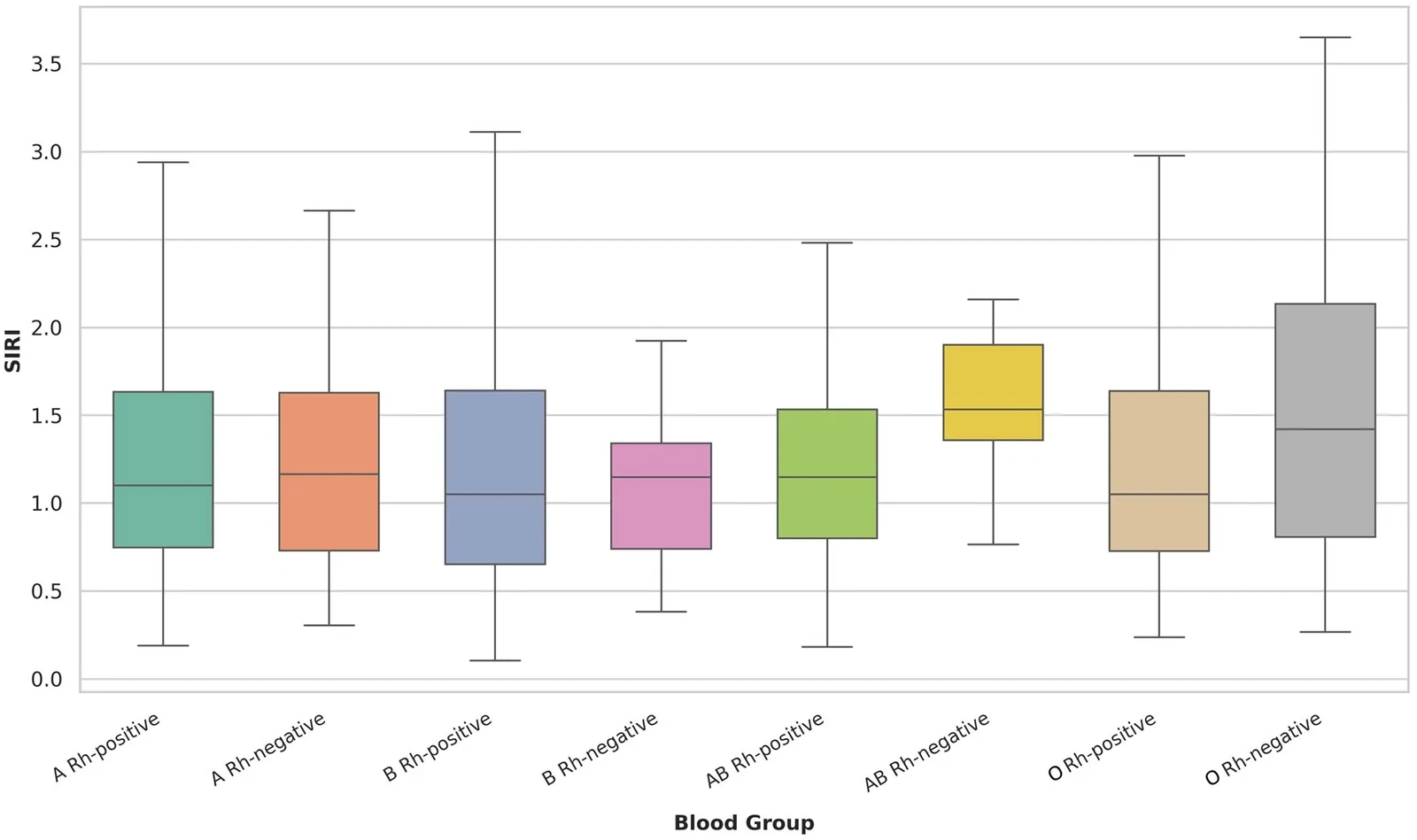
Distribution of the Systemic Inflammation Response Index (SIRI) across ABO and Rh blood group, D antigen (RhD) blood group phenotypes. The box-and-whisker plot displays the comparative spread of baseline SIRI scores across the evaluated blood group cohorts. Median scores, IQRs, and overall nonoutlier distributions are explicitly indicated. Multigroup variation assessment through the Kruskal-Wallis *H* test revealed no statistically significant disparity across the phenotypes (*P* = .498), despite a borderline higher trend observed specifically within RhD-negative subgroups via exploratory independent 2-group analysis (*P* = .06). The index was derived using the mathematical formula (neutrophils × monocytes) / lymphocytes.

**Figure 4:**
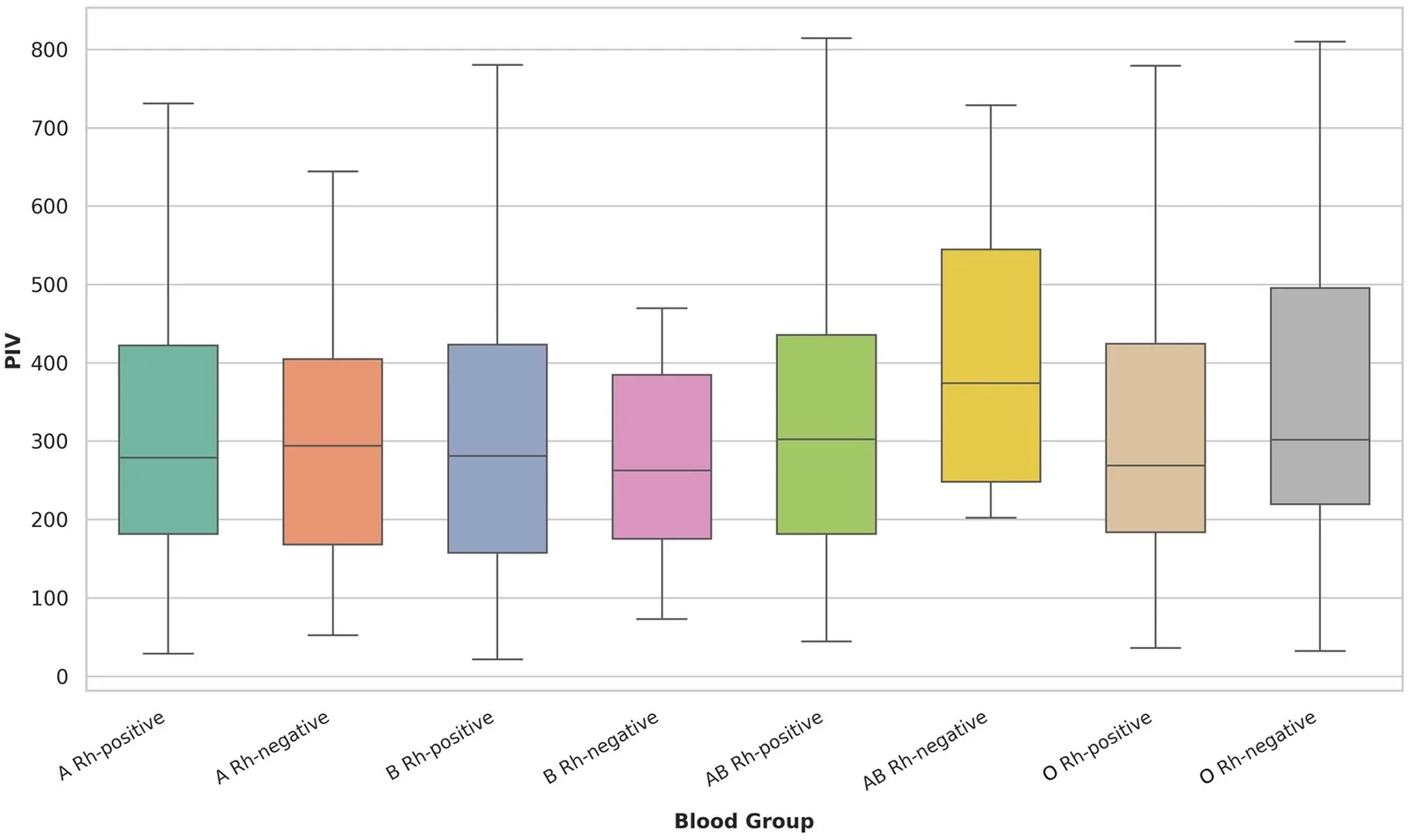
Distribution of the Pan-Immune-Inflammation Value (PIV) across ABO and Rh blood group, D antigen blood group phenotypes. The box-and-whisker plot charts the physiologic stability of the comprehensive multilineage PIV metric across standard blood group profiles. No statistically significant differences or phenotypic fluctuations are present across the clinical cohorts when evaluated using the Kruskal-Wallis *H* test (*P* = .61). The index balances adaptive and innate cellular components according to the integrated formula (neutrophils × platelets × monocytes) / lymphocytes.

Furthermore, phenotypic RhD status did not substantially influence the baseline scores of these novel biomarkers. The independent 2-group analyses revealed no statistically significant disparities between RhD-positive and RhD-negative individuals for SII (*P* = .34), SIRI (*P* = .06), or PIV (*P* = .33). Intriguingly, although RhD-negative participants exhibited a slightly elevated baseline trend in SIRI scores, this variation remained strictly nonsignificant and within the established marginal limits.

### The impact of sex and age on systemic inflammatory indices

In stark contrast to blood group antigens, biological sex emerged as a highly robust and statistically significant determinant for baseline SII and SIRI profiles. A distinct sex-based dimorphism was identified within the healthy cohort: SII scores were statistically significantly elevated in female compared with male individuals (median, 573.68 vs 530.58; *P* = .007). Conversely, SIRI scores were statistically significantly higher in male than in female individuals (median, 1.20 vs 1.06; *P* = .04). No statistically significant sex-specific variations were detected for PIV (median, 280.71 in male individuals vs 279.94 in female individuals; *P* = .61) (Table 1).

When stratified into specific chronological age brackets, multigroup comparisons revealed no statistically significant differences in SII, SIRI, or PIV scores between young adults (aged 18-39 years), middle-aged adults (aged 40-59 years), and older adults (aged ≥60 years; all *P* > .05) (Table 1). Continuous analysis via Spearman rank correlation firmly corroborated these findings, indicating that chronological age did not statistically significantly correlate with SII (*r* *=* *‒*0.012, *P* = .72), SIRI (*r* *=* .031, *P* = .35), or PIV (*r* *=* *‒*0.019, *P* = .56) (Table 1). These cumulative findings robustly indicate that within a strictly screened, metabolically healthy adult population, these composite markers maintain physiologic stability across the adult lifespan.

## Discussion

This study investigated the potential association between ABO blood groups and novel systemic inflammatory indices (SII, SIRI, and PIV) in a large, strictly screened cohort of healthy adults. Our results demonstrate that neither ABO blood group nor RhD status strongly influences these integrated markers of inflammation.

The relationship between the ABO system and systemic inflammation remains a subject of long-standing interest. As noted by Liumbruno et al^10^ and Franchini et al,^25^ ABO antigens are expressed on various cell types, including vascular endothelial cells. Previous studies by Jenkins et al^11^ and Franchini et al^25^ have suggested that ABO antigens are intricately linked to cardiovascular disease and cancer through the modulation of proinflammatory mediators and von Willebrand factor. Despite these established biological links among disease states, our analysis showed that in clinical health, fundamental cellular ratios remain consistent across phenotypes. This finding suggests that although ABO-specific glycosylation may influence disease susceptibility, as discussed by Ward et al,^12^ the baseline inflammatory tonus is not predetermined by the ABO genotype, a finding further contextually supported by the molecular insights of Storry et al.^26^

One of the most striking findings is the substantial impact of sex. The higher SII in female individuals aligns with the findings of Becker et al,^27^ who noted sex-based differences in platelet reactivity. Conversely, the higher SIRI scores in male individuals reflect the androgenic influence on myelopoiesis, as detailed by Oertelt-Prigione.^28^ Interestingly, PIV remained stable across sexes, suggesting a balancing effect within the formula where sex-specific variations in cell counts—noted also by Cheng et al^16^—neutralize each other. These results reinforce the necessity of using sex-stratified reference ranges, as previously identified in large-scale studies by Luo et al,^13^ Fei et al,^23^ and Fest et al.^29^

In addition to sex-based variations, a borderline trend toward higher SIRI score was noted in Rh-negative individuals (*P* = .06). Although this near-significant value hints at potential, subtle microenvironmental discrepancies in myeloid lineages linked to the Rhesus membrane structure, it must be interpreted with caution. The relatively low proportion of Rh-negative participants (14.7%) in our cohort represents a limitation that inherently reduced the statistical power for this specific subanalysis. This finding echoes the power constraints observed in recent regional studies exploring ABO/Rh distributions in transient metabolic conditions such as gestational diabetes^17^^,^^18^ or systemic immunologic evaluations in chronic pathologies such as Behçet disease.^19^ Furthermore, given the retrospective, cross-sectional design of our study and the unavoidable absence of tracking specific lifestyle-related variables (eg, precise physical workloads, micro-dietary variations, hydration patterns), terms such as *strictly healthy* or *true baseline* should be viewed as conceptual approximations rather than definitive biological absolutes.

Regarding age, our study found no statistically significant correlation, which contrasts with broader population studies such as that by García-Domínguez.^30^ This discrepancy is likely attributable to our rigorous exclusion criteria, which isolated a successfully aging population. By strictly excluding participants with metabolic dysregulation, as comprehensively defined in the recent population-based studies by Li X et al,^3^ Li L et al,^20^ and Zhao et al,^4^ we successfully bypassed the confounding effects of age-related systemic inflammatory noise. This finding possibly reflects that successful metabolic preservation and structural organ stability bypass typical age-associated low-grade systemic inflammation (“inflammaging”). However, the initial implication emphasizing metabolic health over chronological age must be interpreted cautiously because longitudinal validation is required to determine whether these indices remain tightly bound to metabolic parameters across the full lifespan. Finally, although Flegel^31^ highlighted the genetic complexity of the Rhesus system, our analysis confirms its negligible impact on basal inflammatory indices.

### Strengths and limitations

This study has several notable strengths. First, the large sample size (*n* = 923) and the rigorous exclusion of individuals with even minor metabolic abnormalities or subclinical dysfunctions (eg, fasting glucose, lipid profiles, thyroid status) ensure that the data represent a truly baseline physiologic state. Unlike many studies that use general population data, our “strictly healthy” cohort allows for a more precise characterization of the inherent influence of blood group antigens, similar to the methodologic precision emphasized by Xu et al.^14^ Second, based on a literature search using the terms 'ABO blood groups', 'systemic immune-inflammation index', 'SIRI', and 'PIV' in PubMed and Web of Science, our study is 1 of the first to simultaneously evaluate SII, SIRI, and PIV in relation to both ABO and RhD phenotypes in a healthy adult population.

However, certain limitations must be acknowledged, as well. First, the retrospective, cross-sectional design prevents the assessment of the long-term stability of these indices within the same individuals over time. Second, although we used rigorous clinical and laboratory exclusion criteria, data on specific lifestyle factors, such as physical activity levels, dietary habits, and hydration status—which could influence minor fluctuations in hematologic parameters—were not available in the electronic health records. Third, the study population was derived from a single tertiary-care center, which may limit the generalizability of the findings to different ethnic or geographic populations, a factor that Fei et al^23^ and Fest et al^29^ have highlighted as important in establishing regional reference intervals.

In conclusion, our study demonstrated that the novel systemic inflammatory indices SII, SIRI, and PIV are independent of ABO blood groups and RhD status in clinically healthy adults. These findings suggest that blood group antigens do not inherently modulate the baseline cellular ratios of the innate and adaptive immune systems. However, the presence of statistically significant sex-specific dimorphism—with higher SII scores in female individuals and higher SIRI scores in male individuals—highlights the necessity of establishing sex-stratified reference intervals for accurate clinical interpretation. Furthermore, the stability of these markers across different age groups in our strictly screened cohort underscores their potential as reliable baseline indicators of systemic health. These results provide a robust foundation for future research that uses these indices as prognostic or diagnostic biomarkers in various pathologic states.

## Data Availability

The data underlying this article will be shared on reasonable request to the corresponding author.

## References

[1] Saylik F , Akbulut T. Systemic Immune-Inflammation Index predicts major cardiovascular adverse events in patients with ST-segment elevated myocardial Infarction. Arq Bras Cardiol. 2022;119:14-22. doi:10.36660/abc.20210412 PMC9352114 35830117

[2] Yaşar E , Bayramoğlu A. Systemic Immune-Inflammation Index as a predictor of microvascular dysfunction in patients with cardiac syndrome X. Angiology. 2022;73:615-621. doi:10.1177/00033197221087777 35403436

[3] Li X , Cui L, Xu H. Association between systemic inflammation response index and chronic kidney disease: a population-based study. Front Endocrinol (Lausanne). 2024;15:1329256. doi:10.3389/fendo.2024.1329256 PMC10917959 38455650

[4] Zhao S , Dong S, Qin Y, Wang Y, Zhang B, Liu A. Inflammation index SIRI is associated with increased all-cause and cardiovascular mortality among patients with hypertension. Front Cardiovasc Med. 2022;9:1066219. doi:10.3389/fcvm.2022.1066219 PMC9874155 36712259

[5] Templeton AJ , McNamara MG, Šeruga B, et al Prognostic role of neutrophil-to-lymphocyte ratio in solid tumors: a systematic review and meta-analysis. J Natl Cancer Inst. 2014;106:dju124. doi:10.1093/jnci/dju124. PMID: 24875653 24875653

[6] Hu B , Yang XR, Xu Y, et al Systemic immune-inflammation index predicts prognosis of patients after curative resection for hepatocellular carcinoma. Clin Cancer Res. 2014;20:6212-6222. doi:10.1158/1078-0432.CCR-14-0442 25271081

[7] Fucà G , Guarini V, Antoniotti C, et al The Pan-Immune-Inflammation Value is a new prognostic biomarker in metastatic colorectal cancer: results from a pooled-analysis of the Valentino and TRIBE first-line trials. Br J Cancer. 2020;123:403-409. doi:10.1038/s41416-020-0894-7 PMC7403416 32424148

[8] Zahorec R. Ratio of neutrophil to lymphocyte counts—rapid and simple parameter of systemic inflammation and stress in critically ill. Bratisl Lek Listy. 2001;102:5-14. doi:10.1007/BF03656390 11723675

[9] Qi Q , Zhuang L, Shen Y, et al A novel Systemic Inflammation Response Index (SIRI) for predicting the survival of patients with pancreatic cancer after chemotherapy. Cancer. 2016;122:2158-2167. doi:10.1002/cncr.30057 27152949

[10] Liumbruno GM , Franchini M. Beyond immunohaematology: the role of the ABO blood group in human diseases. Blood Transfus. 2013;11:491-499. doi:10.2450/2013.0152-13 PMC3827391 24120598

[11] Jenkins PV , O’Donnell JS. ABO blood group determines plasma von Willebrand factor levels: a biologic function after all? Transfusion. 2006;46:1836-1844. doi:10.1111/j.1537-2995.2006.00975.x 17002642

[12] Ward SE , O’Sullivan JM, O’Donnell JS. The relationship between ABO blood group, von Willebrand factor, and primary hemostasis. Blood. 2020;136:2864-2874. doi:10.1182/blood.2020005843 PMC7751360 32785650

[13] Luo H , He L, Zhang G, et al Normal reference intervals of neutrophil-to-lymphocyte ratio, platelet-to-lymphocyte ratio, lymphocyte-to-monocyte ratio, and Systemic Immune Inflammation Index in healthy adults: a large multi-center study from western China. Clin Lab. 2019;65:655-665. doi:10.7754/Clin.Lab.2018.180715 30868857

[14] Xu JP , Zeng RX, Zhang YZ, et al Systemic inflammation markers and the prevalence of hypertension: a NHANES cross-sectional study. Hypertens Res. 2023;46:1009-1019. doi:10.1038/s41440-023-01195-0 36707716

[15] Klein SL , Flanagan KL. Sex differences in immune responses. Nat Rev Immunol. 2016;16:626-638. doi:10.1038/nri.2016.90 27546235

[16] Cheng T , Yu D, Tang Q, et al Gender differences in the relationship between the systemic immune-inflammation index and all-cause and cardiovascular mortality among adults with hypertension: evidence from NHANES 1999-2018. Front Endocrinol (Lausanne). 2024;15:1436999. doi:10.3389/fendo.2024.1436999 PMC11493643 39439560

[17] Keskin A , Aci R, Erdem M, et al ABO blood group phenotypes and Rhesus factor in the pathogenesis of gestational diabetes mellitus. Konuralp Med J. 2024;16:278-282. doi:10.18521/ktd.1525270

[18] Aci R , Keskin ADEM, Bilgin M, Türe EDA. Inflammatory markers and gestational diabetes mellitus risk: investigating neutrophil/lymphocyte ratio, platelet/lymphocyte ratio, and systemic immune inflammation index. Ain Shams Med J. 2024;75:646-651. doi:10.21608/asmj.2024.290292.1273

[19] Aci R , Keskin A, Sezer Ö, et al Evaluation of full blood count–derived inflammatory indices (SII, SIRI, PIV, NLR) in Behçet’s disease patients and healthy controls. OBM Genet. 2025;09:1. doi:10.21926/obm.genet.2504318

[20] Li L , Chen K, Wen C, Ma X, Huang L. Association between Systemic Immune-Inflammation Index and chronic kidney disease: a population-based study. PLoS One. 2024;19:e0292646. doi:10.1371/journal.pone.0292646 PMC10852278 38329961

[21] American Diabetes Association Professional Practice Committee. 2. Diagnosis and classification of diabetes: standards of care in diabetes—2024. Diabetes Care. 2024;47:S20-S42. doi:10.2337/dc24-S002 PMC10725812 38078589

[22] Cappellini MD , Motta I. Anemia in clinical practice—definition and classification: does hemoglobin change with aging? Semin Hematol. 2015;52:261-269. doi:10.1053/j.semminhematol.2015.07.006 26404438

[23] Fei Y , Wang X, Zhang H, Huang M, Chen X, Zhang C. Reference intervals of Systemic Immune-Inflammation Index, neutrophil to lymphocyte ratio, platelet to lymphocyte ratio, mean platelet volume to platelet ratio, mean platelet volume and red blood cell distribution width-standard deviation in healthy Han adults in Wuhan region in central China. Scand J Clin Lab Invest. 2020;80:500-507. doi:10.1080/00365513.2020.1793220 32673141

[24] Mishra P , Pandey CM, Singh U, Gupta A, Sahu C, Keshri A. Descriptive statistics and normality tests for statistical data. Ann Card Anaesth. 2019;22:67-72. doi:10.4103/aca.ACA_157_18 PMC6350423 30648682

[25] Franchini M , Lippi G. The intriguing relationship between the ABO blood group, cardiovascular disease, and cancer. BMC Med. 2015;13:7. doi:10.1186/s12916-014-0250-y PMC4295232 25592962

[26] Storry JR , Olsson ML. The ABO blood group system revisited: a review and update. Immunohematology. 2009;25:48-59. doi:10.21307/immunohematology-2019-231 19927620

[27] Becker DM , Segal J, Vaidya D, et al Sex differences in platelet reactivity and response to low-dose aspirin therapy. JAMA. 2006;295:1420-1427. doi:10.1001/jama.295.12.1420 16551714

[28] Oertelt-Prigione S. The influence of sex and gender on the immune response. Autoimmun Rev. 2012;11:A479-A485. doi:10.1016/j.autrev.2011.11.022 22155201

[29] Fest J , Ruiter R, Ikram MA, Voortman T, van Eijck CHJ, Stricker BH. Reference values for white blood-cell-based inflammatory markers in the Rotterdam Study: a population-based prospective cohort study. Sci Rep. 2018;8:10566. doi:10.1038/s41598-018-28646-w PMC6043609 30002404

[30] García-Domínguez M. Pathological and inflammatory consequences of aging. Biomolecules. 2025;15:404. doi:10.3390/biom15030404 PMC11939965 40149940

[31] Flegel WA. The genetics of the Rhesus blood group system. Blood Transfus. 2007;5:50-57. doi:10.2450/2007.0011-07 PMC2535884 19204754

